# Supplementary material for: Sigma oscillations protect or reinstate motor memory depending on their temporal coordination with slow waves
Source: eLife. 2022 Jun 21;11:e73930. doi: 10.7554/eLife.73930 (PMC9259015; doi:10.7554/eLife.73930)
Supplement: Supplementary file 1. [file elife-73930-supp1.docx]

| **Supplementary file 1.** Sleep and stimulation characteristics (N=24). | | | | | | |
| --- | --- | --- | --- | --- | --- | --- |
| **Daytime sleep characteristics** | | | | | | |
| Time allowed to sleep | 89.7 min [88.5 – 90.9] | |  | |  | |
| Total Sleep Time^a^ | 67 min [61.1 – 73] | |  | |  | |
| Sleep Efficiency^b^ | 74.9% [68.1 –81.8] |  | | |  | |
| NREM1 Latency | 4.5 min [3.4 – 5.6] |  | | |  | |
| Time awake | 10.6 min [6.9 – 14.3] |  | | |  | |
| Time in NREM1 Sleep | 12.1 min [8.6 – 15.5] |  | | |  | |
| Time in NREM2 Sleep | 39.9 min [33.8 – 46.1] | | |  | |  |
| Time in NREM3 Sleep | 17.5 min [8.7 – 26.2] |  | | |  | |
| Time in REM Sleep | 9.6 min [5.8 – 13.4] |  | | |  | |
| **Participants reaching** |  |  | | |  | |
| NREM3 sleep | N = 20 |  | | |  | |
| REM sleep | N = 18 |  | | |  | |
| **Number of Auditory cues** | *All cues* | *Associated cues* | | | *Unassociated cues* | |
| During all stages | 389 [339 – 439] | 192.5 [167.1 – 218.1] | | | 196.4 [171.5 – 221.3] | |
| During wake | 8.8 [4.1 – 13.5] | 3.8 [1.2 – 6.4] | | | 5 [1.8 – 8.2] | |
| During NREM1 Sleep | 22.1 [9.1 – 35.1] | 11.6 [4.7 – 18.5] | | | 10.5 [3.9 – 17.1] | |
| During NREM2-3 Sleep | 349.5 [293.8 – 405.2] | 173.4 [145.3 – 201.5] | | | 176.1 [147.9 – 204.3] | |
| During REM Sleep | 8.5 [3.6 – 13.5] | 3.8 [1 – 6.5] | | | 4.8 [1.7 – 7.9] | |
| Accuracy^c^ | 88.4% [82.9 – 93.9] | 88.7% [82.8 –94.6] | | | 88.2% [82.3 – 94] | |
|  |  | | | | | |
| *Notes*. Values are means [lower and upper limit of the 95% Confidence Interval - CI]. REM: Rapid Eye Movement. ^a^ Total sleep time was computed as the total time spent in stages NREM2-3 and REM sleep. ^b^Sleep efficiency was computed as the percent of time asleep (namely in NREM2-3 and in REM sleep) relative to the total time in bed (specifically, from lights off to lights on). ^c^ Percentage of auditory cues correctly sent during NREM2 and NREM3 sleep. | | | | | | |
