# Supplementary material for: Sigma oscillations protect or reinstate motor memory depending on their temporal coordination with slow waves
Source: eLife. 2022 Jun 21;11:e73930. doi: 10.7554/eLife.73930 (PMC9259015; doi:10.7554/eLife.73930)
Supplement: Supplementary file 2. [file elife-73930-supp2.docx]

**Supplementary file 2**. Mean number [lower and upper limit of the 95% CI] of sleep events across participants detected at the single channel level per condition of blocks (either stimulation associated/unassociated or rest).

|  | Slow waves | | | | |  |
| --- | --- | --- | --- | --- | --- | --- |
|  | | Associated | Unassociated | Rest | Entire Nap |  |
| Fz | | 159.7 [80.3 – 239.1] | 161 [83.3 – 238.8] | 155.5 [88.9 – 222.1] | 469.5 [255.6 - 683.4] |  |
| Cz | | 139 [71.9 – 206.2] | 139.4 [74.5 204.3] | 139 [80.8 – 197.2] | 411.6 [231.4 - 591.7] |  |
| Pz | | 121.3 [58.8 – 183.9] | 115.7 [59.2 – 172.2] | 109.7 [60.9 – 158.7] | 336.9 [178.7 - 495.1] |  |
| Oz | | 52.6 [18.5 – 86.7] | 51.6 [19.8 – 83.5] | 42.8 [20.9 – 64.6] | 130.5 [52.7 - 208.3] |  |
| C3 | | 99.8 [41.3 - 158.3] | 97.2 [41.9 – 152.6] | 91.3 [47.3 – 135.4] | 284.3 [132 - 436.7] |  |
| C4 | | 107.6 [46.2 – 169.1] | 102.2 [46.1 158.2] | 100.5 [52.7 – 148.4] | 297.4 [141.4 - 453.4] |  |
|  | | **Spindles** | | | | |
|  | | Associated | Unassociated | Rest | Entire Nap |  |
| Fz | | 27.7 [14.6 – 40.8] | 31.9 [17.3 – 46.4] | 49.2 [30.7 – 67.6] | 102.6 [62.8 - 142.4] |  |
| Cz | | 28.4 [14.9 – 41.9] | 34.3 [19.8 – 48.9] | 54 [36 – 71.9] | 114.1 [72.5 - 155.7] |  |
| Pz | | 27.5 [14.8 – 40.1] | 34.2 [20.2 – 48.3] | 59 [42 – 75.9] | 119.5 [80.6 - 158.5] |  |
| Oz | | 11.8 [5 – 18.6] | 11.2 [6 – 16.4] | 22.7 [12.1 – 33.3] | 37.5 [19.7 - 55.4] |  |
| C3 | | 25 [13.1 - 37] | 30.6 [16 – 45.2] | 52.4 [34.9 – 69.9] | 105.7 [66.5 - 144.9] |  |
| C4 | | 27 [12.6 – 41.5] | 31 [16.3 – 45.7] | 54.1 [34.8 – 73.3] | 108.5 [65 - 152.1] |  |
