# Supplementary material for: Sigma oscillations protect or reinstate motor memory depending on their temporal coordination with slow waves
Source: eLife. 2022 Jun 21;11:e73930. doi: 10.7554/eLife.73930 (PMC9259015; doi:10.7554/eLife.73930)
Supplement: Supplementary file 3. — These deviations are marked with a # in the main manuscript. [file elife-73930-supp3.docx]

**Supplementary file 3**. List of the deviations from the pre-registered analyses followed by their justification. These deviations are marked with a ^#^ in the main manuscript.

|  | **Pre-registered** | **Final report** |
| --- | --- | --- |
| 1 | Only right-handed participants | Both right- and **left**-handed participants |
|  | **Justification**: Based on the bimanual nature of our motor task, we elected to not restrict our participant pool to only right-handers in order to facilitate recruitment (N= 2 left-handers). | |
| 2 | Pre-nap performance for offline gain computation on last 4 blocks of the MSL | Pre-nap performance for offline gain computation on last **3** blocks of the MSL |
|  | Justification: See main manuscript for details. Briefly, against our expectations based on previous research using learning of a single sequence, participants only reached plateau performance on the two sequences starting on block 2 of the pre-nap test. In order to meet the performance plateau pre-requisite to compute offline gains in performance, we therefore excluded the first block of the pre-nap test and computed offline gains based on the last 3 blocks of the pre-nap test which showed stable performance levels for both sequences. | |
| 3 | We will classify auditory evoked responses into evoked SO if the standard criteria of a SO are met (negative peak ≤ -40 µV and the peak-to-peak amplitude ≥ 75 µV). Mean auditory-evoked SO amplitude will be computed for each subject in each condition separately. | Auditory-evoked responses were averaged **across all trials** for each condition. Mean auditory **ERP** amplitude was computed for each subject in each condition separately. |
|  | Justification: The number of auditory ERPs reaching the pre-registered amplitude criteria was not sufficient to perform a powerful statistical analysis. This issue being highlighted in previous research (4), we followed similar procedures and averaged all the auditory-evoked responses (irrespective of their amplitude) on the one hand and all the detected SO on the other hand (4). | |
| 4 | To analyze spindle activity, we will compute Time-Frequency representations (TFR) using Morlet Wavelet decomposition with a width of five cycles per wavelet (m=7) at center frequencies between 12 and 16 Hz (sigma frequency band), in steps of 0.5 Hz and 10 ms. | To analyze spindle activity, we computed Time-Frequency representations (TFR) using **an adaptive sliding time window of five cycles length per frequency (Δt = 5/f; 20-ms step size), and estimated power using the Hanning taper/FFT approach between 5 and 30 Hz**. |
|  | Justification: For completeness, we decided to broaden our analysis from 12-16 Hz to 5-30 Hz. Consequently, the Morlet wavelet decomposition approach was not appropriate to capture low (around 5 Hz) frequency band in regards of the epoch size. Note that any effects observed outside the pre-registered frequency band (i.e, sigma) are reported in the main text as the result of exploratory analyses. A similar frequency window approach was used for the PAC analyses. | |
| 5 | The mean of the circular angle values for SO-spindle coupling will be compared between stimulation blocks of associated vs. unassociated auditory cues using a one-tailed paired student t test. | The mean of the circular angle values for SO-spindle coupling was compared between stimulation blocks of associated vs. unassociated auditory cues using **the CircStat toolbox (24) implementing Rayleigh test for non-uniformity and Watson-Williams multi-sample test for equal means** |
|  | Justification: This statistical test was more appropriate for circular data as compared to the classical student t test (5). | |
| 6 | Parametric statistical testing will be applied, namely Student t-tests for comparison of means and Pearson tests for correlation analyses. | We performed non-parametric statistical testing whenever the normality criterion was not met, **namely Wilcoxon signed-rank tests for comparison of means and Spearman tests for correlation analyses.** |
|  | Justification: The assumption of normality was not met for some metrics; we thus used the equivalent non-parametric test in these cases. | |
| 7 | Pearson correlations will be performed between the TMR index and the relative change between sigma band power time-locked to the associated auditory cues and the sigma band power time-locked to unassociated auditory cues. | The correlation between TMR index and the difference between sigma band power time-locked to the associated auditory cues and the sigma band power time-locked to unassociated auditory cues was performed using **CBP tests due to the time-frequency dimension of the data.** |
|  | Justification: Correlation analyses between the TMR index and the raw difference in sigma band power time-locked to the associated and unassociated auditory cues were performed using Cluster Based permutation tests (6). This approach was preferred as it is more conservative than the analysis initially pre-registered and allows to handle and correct for the large time-frequency dimension of the data. Accordingly, CBP approaches were also used for the correlation analyses between PAC and TMR index. | |
