# Supplementary material for: Sigma oscillations protect or reinstate motor memory depending on their temporal coordination with slow waves
Source: eLife. 2022 Jun 21;11:e73930. doi: 10.7554/eLife.73930 (PMC9259015; doi:10.7554/eLife.73930)
Supplement: Transparent reporting form [file elife-73930-transrepform1.pdf]

eLIFE
1st Floor
24 Hills Road
Cambridge CB2 1JP, UK
P 01223 855340
W elifesciences.org
T @elife

eLife's transparent reporting form

We encourage authors to provide detailed information *within their submission* to facilitate the interpretation and replication of experiments. Authors can upload supporting documentation to indicate the use of appropriate reporting guidelines for health-related research (see EQUATOR Network), life science research (see the BioSharing Information Resource), or the ARRIVE guidelines for reporting work involving animal research. Where applicable, authors should refer to any relevant reporting standards documents in this form.

If you have any questions, please consult our Journal Policies and/or contact us: editorial@elifesciences.org.

Sample-size estimation

- You should state whether an appropriate sample size was computed when the study was being designed
- You should state the statistical method of sample size computation and any required assumptions
- If no explicit power analysis was used, you should describe how you decided what sample (replicate) size (number) to use

Please outline where this information can be found within the submission (e.g., sections or figure legends), or explain why this information doesn't apply to your submission:

The sample size estimation is thoroughly described in the pre-registered report (available at osf.io/n3me8) and in the supplmentary document (page 1, first paragraph of Supplementary material & methods section)

Replicates

- You should report how often each experiment was performed
- You should include a definition of biological versus technical replication
- The data obtained should be provided and sufficient information should be provided to indicate the number of independent biological and/or technical replicates
- If you encountered any outliers, you should describe how these were handled
- Criteria for exclusion/inclusion of data should be clearly stated
- High-throughput sequence data should be uploaded before submission, with a private link for reviewers provided (these are available from both GEO and ArrayExpress)

Please outline where this information can be found within the submission (e.g., sections or figure legends), or explain why this information doesn't apply to your submission:

The number of all replicates are given in the section 4.1 (Participants, Materials and Methods). OUtliers handling is described both in the pre-registered report and in the section Preprocessing of Behavioral data in the Materials and Methods (4.5.1.1). Criteria for exclusion/inclusion hve been declared in the pre-registered report and in the section 4.1 (Participants, Materials and Methods).

eLife Sciences Publications, Ltd is a limited liability non-profit non-stock corporation incorporated in the State of Delaware, USA, with company number 5030732, and is registered in the UK with company number FC030576 and branch number BR015634 at the address 1st Floor, 24 Hills Road, Cambridge CB2 1JP | August 2014

eLIFE
1st Floor
24 Hills Road
Cambridge CB2 1JP, UK
P 01223 855340
W elifesciences.org
T @elife

Statistical reporting

- Statistical analysis methods should be described and justified
- Raw data should be presented in figures whenever informative to do so (typically when N per group is less than 10)
- For each experiment, you should identify the statistical tests used, exact values of N, definitions of center, methods of multiple test correction, and dispersion and precision measures (e.g., mean, median, SD, SEM, confidence intervals; and, for the major substantive results, a measure of effect size (e.g., Pearson's r, Cohen's d)
- Report exact p-values wherever possible alongside the summary statistics and 95% confidence intervals. These should be reported for all key questions and not only when the p-value is less than 0.05.

Please outline where this information can be found within the submission (e.g., sections or figure legends), or explain why this information doesn't apply to your submission:

General information on data analysis given at end of Methods section. Every figure legend has statistical information concerning the data representation, and details of tests. Box and whisker plots (with mean and median) have been used in most cases, except for event related potentials and Slow Waves grand average (SEM is presented) and for Time-Frequency representations. F, t and V (or W) statistics and corrected p-values were reported for ANOVAs, student and Wilcoxon tests, respectively. Effect sizes were reported for significant comparisons using Cohen's d for Student t-tests, r for Wilcoxon signed-rank test and η^2 for rmANOVAs using G*power

(For large datasets, or papers with a very large number of statistical tests, you may upload a single table file with tests, Ns, etc., with reference to sections in the manuscript.)

Group allocation

- Indicate how samples were allocated into experimental groups (in the case of clinical studies, please specify allocation to treatment method); if randomization was used, please also state if restricted randomization was applied
- Indicate if masking was used during group allocation, data collection and/or data analysis

Please outline where this information can be found within the submission (e.g., sections or figure legends), or explain why this information doesn't apply to your submission:

Randomisation was described in the pre-registered report (available at osf.io/n3me8). Each of the two sequences (A and B) will be composed of a particular order of 8 key presses and will be identical for all the subjects. However, the association between the sound (either sound 1, sound 2, or sound 3) and the sequences will be randomized as well as the reactivated sequence (A or B). Thus, 12 groups will be constituted corresponding to the different possibilities of the association (Table 1).

Additional data files ("source data")

- We encourage you to upload relevant additional data files, such as numerical data that are represented as a graph in a figure, or as a summary table
- Where provided, these should be in the most useful format, and they can be uploaded as "Source data" files linked to a main figure or table
- Include model definition files including the full list of parameters used
- Include code used for data analysis (e.g., R, MatLab)
- Avoid stating that data files are "available upon request"

Please indicate the figures or tables for which source data files have been provided:

As stated in the main manuscript, data and all custom scripts will be made publicly available upon publication.
